# Regional Scale High Resolution δ^18^O Prediction in Precipitation Using MODIS EVI

**DOI:** 10.1371/journal.pone.0045496

**Published:** 2012-09-19

**Authors:** Wei-Ping Chan, Hsiao-Wei Yuan, Cho-Ying Huang, Chung-Ho Wang, Shou-De Lin, Yi-Chen Lo, Bo-Wen Huang, Kent A. Hatch, Hau-Jie Shiu, Cheng-Feng You, Yuan-Mou Chang, Sheng-Feng Shen

**Affiliations:** 1 Biodiversity Research Center, Academia Sinica, Taipei, Taiwan; 2 School of Forestry and Resource Conservation, National Taiwan University, Taipei, Taiwan; 3 Department of Geography, National Taiwan University, Taipei, Taiwan; 4 Institute of Earth Sciences, Academia Sinica, Taipei, Taiwan; 5 Department of Computer Science and Information Engineering, National Taiwan University, Taipei, Taiwan; 6 Taiwan Air Force Weather Wing, Taipei, Taiwan; 7 Biology Department, Charles William Post Campus of Long Island University, Brookville, New York, United States of America; 8 Department of Ecoscience and Ecotechnology, National University of Tainan, Tainan, Taiwan; 9 Department of Earth Science, National Cheng Kung University, Tainan, Taiwan; University of Oxford, United Kingdom

## Abstract

The natural variation in stable water isotope ratio data, also known as water isoscape, is a spatiotemporal fingerprint and a powerful natural tracer that has been widely applied in disciplines as diverse as hydrology, paleoclimatology, ecology and forensic investigation. Although much effort has been devoted to developing a predictive water isoscape model, it remains a central challenge for scientists to generate high accuracy, fine scale spatiotemporal water isoscape prediction. Here we develop a novel approach of using the MODIS-EVI (the Moderate Resolution Imagining Spectroradiometer-Enhanced Vegetation Index), to predict δ^18^O in precipitation at the regional scale. Using a structural equation model, we show that the EVI and precipitated δ^18^O are highly correlated and thus the EVI is a good predictor of precipitated δ^18^O. We then test the predictability of our EVI-δ^18^O model and demonstrate that our approach can provide high accuracy with fine spatial (250×250 m) and temporal (16 days) scale δ^18^O predictions (annual and monthly predictabilities [*r*] are 0.96 and 0.80, respectively). We conclude the merging of the EVI and δ^18^O in precipitation can greatly extend the spatial and temporal data availability and thus enhance the applicability for both the EVI and water isoscape.

## Introduction

The water isoscape, the natural variation in stable water isotope ratios [1], [2], has been an important tool in disciplines as diverse as hydrology [1], [3], paleoclimatology [2], ecology [4] and forensic investigation [5]. However, the major limitation of applying isotopic technology is the low density and poor continuity of spatial datasets over multiple year time scales [6]. An important approach to resolve this limitation is to develop space-time-explicit predictive models of water isotope distributions [7], [8]. Existing water isoscape models are almost all based on modeling hydrological cycles, which have greatly enhanced our understanding of isotopic physics and help in predicting large-scale water isoscapes [9], [10]. Nevertheless, complex hydrological processes, such as water transport, exchange, and cloud process, almost inevitably decrease the predictability of these models. Thus, these complexities limit application of the hydrological modeling approach to generate water isoscape values at high accuracy and fine scales [11]. The critical challenge of improving predictability of an isoscape model remains the central focus for isotopic research.

**Figure 1:**
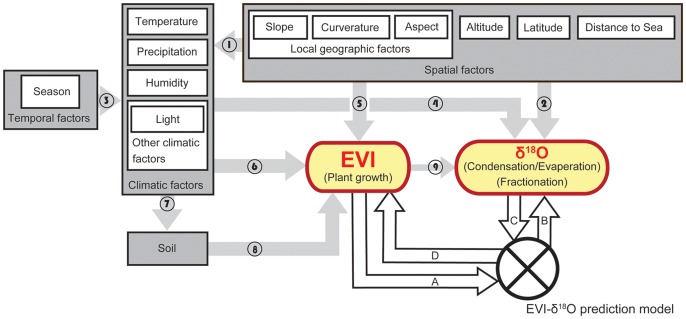
The conceptual basis of merging the EVI and δ^18^O. The direction of influence of each factor is represented by the arrows. Functions that are directly influenced by spatial and climatic factors are listed in the parentheses within the EVI and δ^18^O boxes. “A” is the constraint of spatial extent for EVI data, where is restricted by the amount of snow cover. “B” is the description of the characteristics of output (δ^18^O) including high resolution, large spatial scale, and various temporal scales (16-day, monthly, seasonal or yearly estimation). “C” is the limitation of δ^18^O data. It should be monthly data or the model should be modified to a different temporal scale. “D” indicates the long temporal extent of output of the EVI data. Only temperature, precipitation, and altitude are included in our SEM analysis but other topographic and climatic factors may also be included. References for each relationship is as follows (These references are listed in **Text S1**): [3]–[7] [1], [8]–[10] [2], [9], [11] [3], [8], [10], [12] [5], [13]–[17] [18]–[20] [6], [21], [22] [13], [15], [22] [23]–[25].

Here, we develop a novel approach to predict the precipitated water isoscape. Instead of modeling the hydrological cycle, we used the Enhanced Vegetation Index (EVI), which is a remotely sensed data product of the Moderate Resolution Imagining Spectroradiometer (MODIS) on board the National Aeronautics and Space Administration’s (NASA’s) Terra satellite (MOD13Q1), to estimate the precipitated water isoscape with high spatial and temporal accuracy. The EVI is also an important multidisciplinary tool and can be a surrogate for plant growth [12] or its related biophyscial attribuets such as leaf area index (LAI) [13] in areas with minimum amount of snow cover through time; it is designed for monitoring a wide range of vegetation types (e.g., coniferous and broadleaved/deciduous and evergreen forests) at the global scale [13], which are highly affected by climatic regimes and topography. Similarly, it has also been well-known that the precipitated water isoscape is also influenced by these same factors [14] (**Fig. 1**). Thus, we propose to link these two well-established, but previously separated fields. This novel approach will enable us to predict precipitated δ^18^O (referred as δ^18^O hereafter, see Materials and Methods for details) by using the easy-access EVI data without explicit knowledge of various climatic and topographic data needed by previous isotopic models.

In addition to the predictive model, we also used the structural equation model (SEM), which includes factor analysis, path analysis and regression [15], to explore how climatic and topographic factors influence the integrated EVI (the iEVI, the growing season integrated EVI, measured as the area under the EVI series.) and precipitated δ^18^O through time.

## Materials and Methods

### A. Data Collection

**Figure 2:**
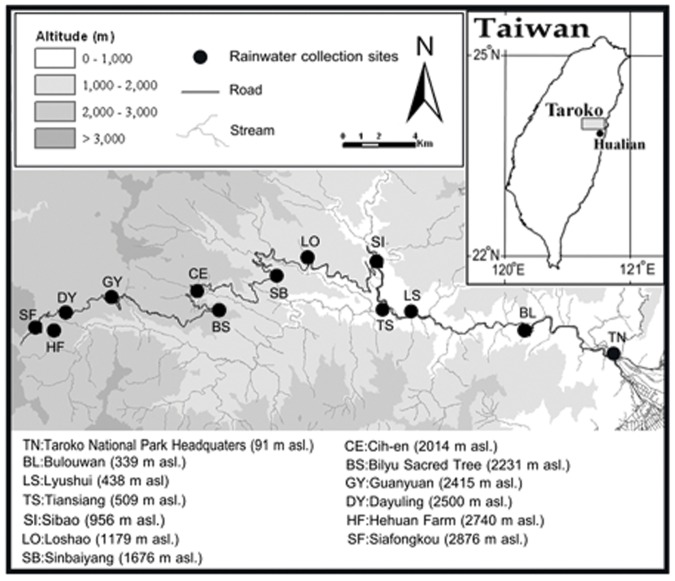
The precipitation collection site. Thirteen precipitation collection sites along an elevation gradient in the eastern Taiwan.

The water samples were collected along an elevational transect in the central Taiwan, from Taroko to Wulin (22°–24°N, 120°–122°E, **Fig. 2**) by the precipitation collected facility (PCF) [16] approximately once a month from April 2007 to December 2009. The data were collected at Lishan [17] (crop land, 121°15′1.5″E, 24°15′32.83″N, 1684 m a.s.l.) is from June, 2003 to May, 2004 and Piluchi (forest, 121°18′28.49″E, 24°13′34.02″N, 2363 m a.s.l.) is from December, 2008 to November, 2009). To prevent the effect caused by the heavy rainfalls mainly induced by tropical cyclones, we collected the water sample right before and after the events. The PCF was composed by a dark glass bottle (1500 c.c.) with 2 cm high mineral oil inside to block the evaporation of collected water, and a plastic funnel (diameter is 6 cm) at the top of the bottle with lace curtain covered to avoid litters. The PCFs were tied at the open area, which have no cover above the funnel [16]. The amounts of samples were first measured in a cylinder (500 c.c.) and recorded as amount of precipitation. We stored samples in a 4°C freezer until analyzing.

Water samples were treated by using CO_2_-H_2_O balancing method [18] and Zinc reduction method [19] and measured in a VG SIRA 10 isotope ratio mass spectrometer to obtain δ^18^O value and δD value [20] (**Fig. S1**).

The EVI data was derived from MODIS observations, produced every 16 days, and downloaded from the NASA website from 2006 to 2010. The hourly temperature data was collected along the elevation transect by the weather stations, which were located in Fushi (109 m), Tiansiang (550 m), Lishan (1980 m), Dayuling (2365 m), Hehuan Mountain (3292 m).

### B. Data Preprocessing

Isotopes measurements and the amount of precipitation occurred in the same temporal scale. The 16-day EVI values (23 sets of data per year) were extracted using ENVI v. 4.6.1 (ITT Visual Information Solutions, Boulder, Colorado, USA). Each site has 23 values a year. Including each value at the middle of the 16 days, and using the “Savitzky-Golay Spline Estimation” function of TIMESAT [21], where the number of envelope iteration was 3, the Adaption Strength is 3, the Savitzky-Golay window size was 5 and the season end is 0.167 (1/6). The iEVI is the growing season integrated EVI, measured as the area under the EVI series, starting from the one sixth of the growing season to the end of the growing season (**Fig. S2**, For the iEVI calculation, see also Mendez-Barroso *et al.*(2009) [22]). In addition, extrema in the data would cause highly uncertainties in prediction along temporal dimensions, so we excluded extrema in the data when comparing predictability (<1 data per season was excluded). Finally, we integrated the EVI to the same temporal scale as δ^18^O data. The quadratic formula prediction was obtained by using Wolfram Mathematica 8 [23]. All data with variances are presented as means ± standard error.

### C. To Obtain the Optimal EVI-δ^18^O Function

Our data is predicted by Wolfram Mathematica 8 with the function:
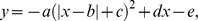
and analyzed in SPSS 18. The sample code of the function to obtain the parameters of the iEVI-δ^18^O function is proved below.

Parallelize[

 evidatan = 20;

 times = 30;

 Timing[

  For[j = 1, j < =  evidatan, j++, {

   singleresult =  ConstantArray[0, {times, 7}];

   result  =  ConstantArray[0, 63];

   t = 1;

   fit  =  ConstantArray[0, evidatan];

   inputxx  =  Import[“c:/users/……/data.xls”][[1, All, j]];

   inputyy  =  Import[“c:/users/……/data.xls”][[1, All, evidatan + j]];

   inputx  =  Drop[inputxx, -Count[inputxx, “na”]];

   inputy  =  Drop[inputyy, -Count[inputyy, “na”]];

   data  =  ConstantArray[0, Length[inputx]];

   Do[{

    Do[{

     Do[{

     For[k = 1, k < =  times, k++, {

      For[i = 1, i < =  Length[inputx], i++, data  =  ReplacePart[

       data, {i} −> {inputx[[i]], inputy[[i]]}]];

      model  =  −a (Abs[x - b] + c)^∧^2+ d x - e;

      Remove[a, b, c, d, e];

      fit  =  FindFit[

       data, {model, {ca < a, 20< b <300, −200< c <0,

        cdmin < d < cdmax, 0< e <100}}, {a, b, c, d, e}, x];

      rr  =  {a, b, c, d, e}/. fit;

      rcy  =  ConstantArray[0, Length[inputx]];

      For[m  = 1, m < =  Length[inputx], m++,

       {xx  =  Extract[inputx, {m}],

        rcy  =  ReplacePart[rcy,

         m −> −rr[[1]] (Abs[xx - rr[[2]]] + rr[[3]])^∧^2+

          rr[[4]] xx - rr[[5]]]}];

      rms  =  Sqrt[Accumulate[(rcy - inputy)^∧^2][[Length[inputx]]]];

      singleresult  =  ReplacePart[singleresult, k -> {rr[[1]], rr[[2]], rr[[3]], rr[[4]], rr[[5]], Correlation[rcy, inputy]^∧^2, rms}];

       }];

      result  = 

       ReplacePart[result, t -> SortBy[singleresult, Last][[1]]];

      t  =  t +1;

      }, {cdmax, 0.1, 0.3, 0.1}]

     }, {cdmin, −0.1, −0.3, −0.1}]

    }, {ca, 0.0005, 0.0035, 0.0005}]

   Print[SortBy[result, Last][[1]]];

  }]

 ]

]

Where the factor range need to be limited. a>0.0005, 20<b<300, −200<c<0, −0.4<d<0.4, 0<e<100. The data.xls format need to be:

E_(i, j)_ E_(i+1, j)_……O_(k, j)_ O_(k+1, j)._

E_(I, j+1)_ E_(i+1, j+1)_……O_(k, j+1)_ O_(k+1, j+1)._

E_(I, j+2)_ E_(i+1, j+2)_……O_(k, j+2)_ O_(k+1, j+2)._

If the length of the data lines are not the same, use “na” to fill all the blank area of the data to make sure all data lines are same length. The parameter “evidatan” required the number of lines (k-1) of the EVI data, and the parameter “times” required the times that Mathematica to find the fitted parameter value. We suggest 30 as the optimal number.

### D. To Make Predictions by the EVI-δ^18^O Function

The code to obtain the parameters of the iEVI-δ^18^O formula is provided below.

evidatan  =  Length[Import[“c:/users/……/constant.xls”][[1, All, 1]]];

For[j  = 1, j < =  evidatan, j++, {

 fit  =  ConstantArray[0, eendline];

 inputxxx  =  Import[“c:/users/……/data.xls””][[1, All, j]];

 inputxx  =  Drop[inputxxx, -Count[inputxxx, “na”]];

 constant  =  Import[“c:/users/……/constant.xls ”][[1, j, All]];

 rcy  =  ConstantArray[0, Length[inputxx]];

 For[m = 1, m < =  Length[inputxx], m++, {xx  =  Extract[inputxx, {m}],

  rcy  =  ReplacePart[rcy, m −> -constant[[1]] (Abs[xx - constant[[2]]] + constant[[3]])^∧^2+ constant[[4]] xx - constant[[5]]]}];

 Print[rcy];}]

  The format of data.xls is just as the description above. The format of constant.xls is:



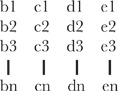



Where n is equal to the “evidatan”, the number of lines (k-1) of the EVI data. It is important that all of the blank areas of the table are count as {} in Mathematica 8. Consequently, to remove all these blanks is crucial. The value of the parameter b of any given time needs to be adjusted by the following formula:

b_predict_ = (b_model_/iEVI_complete growing season of model_)* iEVI_complete growing season of prediction._

Where b_predict_ represents the parameter b, which needs to be input in prediction model, b_model_ represents the most fitted value of parameter b (output from Mathematica) when building the model, the iEVI_complete growing season of model_ represent the data used to construct the model and the iEVI_complete growing season of prediction_ represent the iEVI in the growing season needs to be predicted.

### E. The Stability of the EVI and δ18O

In natural settings, the stabilities of temporal EVI and δ^18^O are depicted as the standard deviation of the four-year time-series EVI and δ^18^O data, respectively. Throughout the result section, p represents the probability of obtaining a test statistic at least as extreme as the one that was actually observed and r represents correlation coefficient, which provides the strength and direction of the dependency between two sets of data.

## Results

### A. The Relationship among Climatic, Topographic Factors, the iEVI and δ^18^O

**Figure 3:**
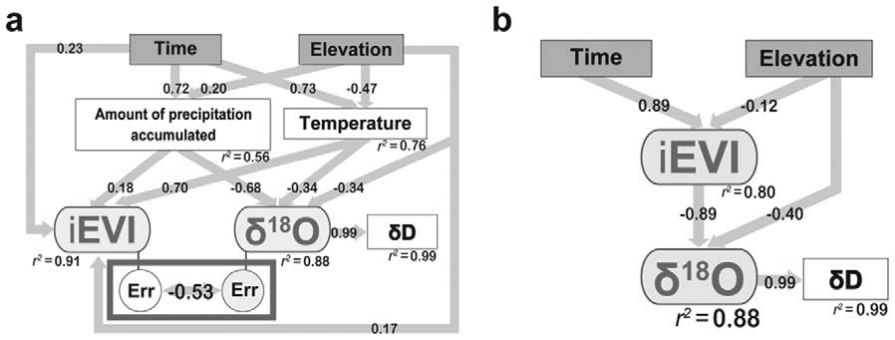
SEM analysis of the relationship among climatic, topographic factors, the iEVI and δ^18^O. **a,** The relationships among the iEVI and δ^18^O and other climatic, topographic factors. The “Err” circles represent the parts that were not explained by above factors in both the iEVI and δ^18^O, but can be explained by each other. **b,** The relationship between topographic factors, the iEVI and δ^18^O (the climatic factors are replaced by the iEVI). All values are under two tailed t-test, and are significant (*p*<0.001). The values shown here are correlation coefficients (*r*), unless noted as *r^2^* representing the overall variability explained by other factors that connect to the box.

Using water samples for δ^18^O analysis collected from 13 sites along the elevation gradient (91–2876 m above sea level [a.s.l.]) in central Taiwan from April 2007 to September 2009 (**Fig. 2**), we found that the spatiotemporal pattern of δ^18^O was highly correlated with the iEVI. Our SEM analysis (n = 403) showed that the effects of precipitation and temperature were pronounced (*p*<0.001) for both the iEVI (*r* = 0.18 and 0.7, respectively) and δ^18^O (*r* = −0.68 and −0.34, respectively) (**Fig. 3a**). Furthermore, 91% and 88% (*p*<0.001) of the iEVI and precipitated δ^18^O can be explained by the climatic and topographic factors, respectively. Additionally, the parts of the iEVI and δ^18^O that were not explained by precipitation, temperature, time and elevation were also highly correlated (*r* = −0.53, *p*<0.001, **Fig. 3a**). Using the same analysis but substituting the climatic factors with the iEVI, the δ^18^O can still explain 88% of variation (**Fig. 3b, ***p*<0.001, n = 403). Moreover, there is a positive linear relationship between the variability of temporal EVI and δ^18^O (*r* = 0.9, *p*<0.001, **Materials and methods E, Fig. S3**). Therefore, the results suggest that the iEVI should be a significant variable for predicting δ^18^O.

### B. Using the iEVI to predict δ^18^O

We further developed a predictive function of using iEVI to predict δ^18^O (referred as iEVI-δ^18^O function, hereafter):
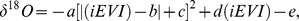
where a, b, c, d and e are constants and vary in different sites, **Table 1**). Thus, even EVI values are the same among different sites at a given time period, the predicted δ^18^O values might still be different since their iEVI-δ^18^O functions are different. We provide a standard procedure of using this function and data from a single growing season to find the predictive function (i.e. find different constants) in each site (**Fig. S4** for the summary of model construction). As a consequence, we can predict continuous time-explicit (every 16 days) δ^18^O values for other seasons in the same region at a spatial resolution of 250 m.

**Table 1:** Constants table.

		Constant				
	Station No.	a	b	c	d	e
Regional stations	s1	0.003001	110.655	−74.069	−0.00393	2.66119
	s2	0.003	121.534	−79.0814	−0.00713	2.65982
	s3	0.003	118.662	−80.6478	−0.00697	3.63887
	s4	0.003015	103.596	−83.0334	0.01662	3.71285
	s5	0.001594	129.2	−107.567	0.005059	4.44664
	s6	0.002052	128.926	−94.7936	−0.00276	4.91114
	s7	0.002	102.816	−107.441	0.01834	4.19058
	s8	0.002079	110.508	−94.6267	−0.00237	5.8392
	s9	0.003	102.438	−84.1505	0.000375	6.07942
	s10	0.002425	101.434	−78.0701	0.008645	6.52685
	s11	0.003719	85.6215	−62.2407	−0.00175	6.66702
	s12	0.003012	83.0063	−67.4471	0.020753	7.81705
	s13	0.004665	71.4633	−54.9761	0.027233	8.55642

Constants calculated from the δ^18^O predictive model for each site (**Fig. 2**).

### C. Predictability of the iEVI-δ^18^O Functions

We used the δ^18^O data collected in Taiwan with the iEVI data in 2007 to develop the iEVI-δ^18^O functions (only four pairs of the iEVI-δ^18^O data were used, one was in the beginning, two were in the middle, and one was at the end of a given growing season) and used the iEVI data to predict δ^18^O in 2008 and 2009. We then utilized this function to predict annual or seasonal δ^18^O for different year(s). By replicating this method, we then cross-validated our models also yielding high (*p*<0.001) annual (*r* = 0.96), seasonal (*r* = 0.96) and monthly (*r* = 0.80±0.17) predictabilities (**Table S1**). High predictabilities (*r* ≥ 0.84) were also observed in other land cover types such as crop land and forest (**Materials and methods A**).

The iEVI-δ^18^O functions can provide not only accurate annual/monthly δ^18^O as described above, but within year δ^18^O estimate. The iEVI-δ^18^O functions were developed using only four-pairs of the iEVI-δ^18^O data to predict the within-season monthly δ^18^O value, and the model was robust (*r* = 0.95±0.01, *p*<0.001, **Table S1**). Note that the predictability of models was slightly weakened (*r* ≥ 0.54, *p*<0.001) during the summer monsoon season (July-September) mainly due to episodic heavy rainfalls presumably induced by tropical cyclones.

**Figure 4:**
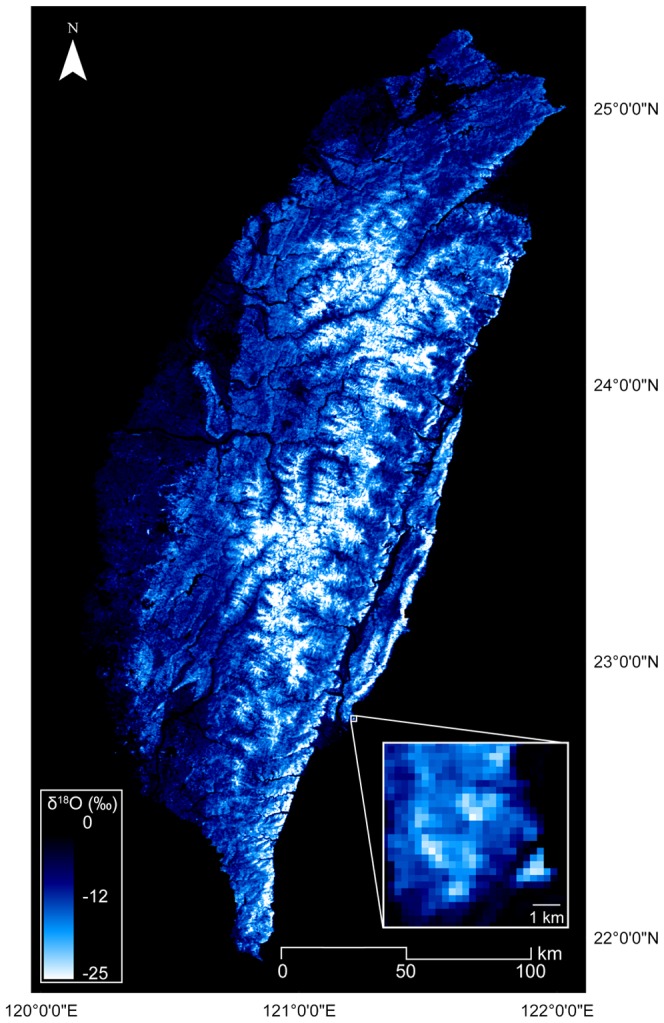
The fine scale regional δ^18^O prediction map of Taiwan in August, 2009. The inset highlights the fine resolution (250 m) of modeled result.

For finer scale spatial δ^18^O estimation, we found that the predictability of the iEVI-δ^18^O functions was significantly higher when using focal EVI values compared to using EVI values in surrounding grids (mean difference in predictability [*r*] for using focal grids and (1) grids 250 meter away [56 grids]: −0.0098±0.00305, *p* = 0.002, and (2) grids 500 meters away [112 grids]: −0.0070±0.00229, *p* = 0.003). This justified the use of MODIS EVI for high resolution mapping of δ^18^O. Given the high spatial predictability of our functions, we can use a single season’s data set for three elevations (91, 1676 and 2876 m a.s.l.) to interpolate the δ^18^O of other sites with high accuracy (*p*<0.001) along the elevation gradient at the regional scale (annual prediction with *r* = 0.96 and monthly prediction with *r* = 0.77±0.00, **Figs. 3** and **S5**, **S6**). Thus, we can use the iEVI-δ^18^O relationship and the δ^18^O-topography relationship to generate a detailed regional scale (250 m×250 m) δ^18^O isoscape (**Fig. 4**).

**Figure 5:**
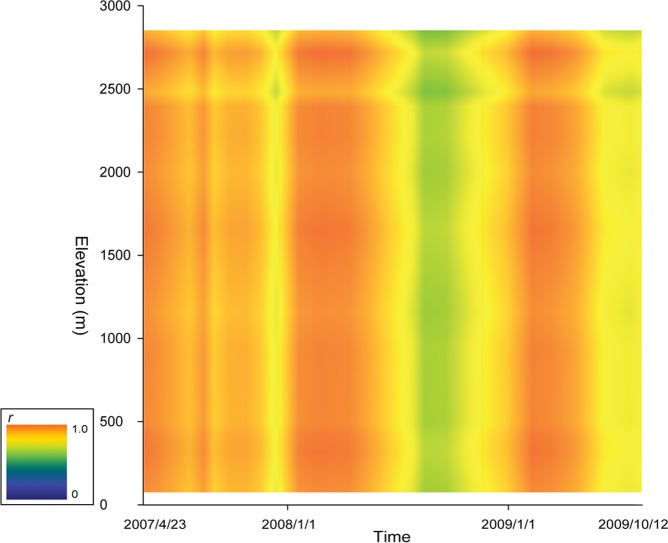
The fine spatiotemporal scale predictability of the model. The predictability in continuous elevation and time data is shown. Using one-growing-season the iEVI-δ^18^O function at Taroko (91 m a.s.l.), Sinbaiyang (1676 m) and Siafongkuo (2876 m) and the δ^18^O-altitude relationship to predict the δ^18^O values along the elevation gradient and in other growing seasons.

## Discussion

Since the EVI is available at the global scale with 16-day sampling intervals, our approach in using the EVI to predict the water isoscape can potentially increase the accessibility of water isotope data in both spatial and temporal dimensions. The previously developed models are especially useful in improving our climatological understanding of isotope distributions at the global scale [24], [25]. Most of these existing models can also be applied to provide long-term averaged climatological predictions at the monthly or seasonal time scales at high spatial (km or sub-km) resolutions where appropriate gridded input data are available. However, small scale hydrological phenomena could not be easily integrated into the global model [26], [27] and, similarly, taking local topographic attributes into account is computationally costly for predicting the global water isoscape [11]. These limitations might be the reasons that existing models have not been applied to finer spatial and temporal scale predictions.

To our best knowledge, our model is the first model providing high accuracy spatial predictions along different altitudes at monthly (calendar month) time scale (**Fig. 5**). In addition, our model also produced higher resolution (250 m×250 m) water isoscape predictions and accuracy than all the existing models in all temporal scales. Therefore, we believe the proposed approach can further enhance the application of the water isotope data and analyses, especially in cases where fine scale and high accuracy isoscape data are important, such as in hydrology [1], [3], forensic investigations [5] and animal migration research [28]. Different from the existing isoscape models that are based on modeling the hydrological cycle, our model can obtain better prediction with minimal efforts of precipitated δ^18^O sampling.

Discovery of a strong relationship between the EVI and δ^18^O also permits potential research directions. For instance, the EVI is only available since year 2000 and thus mainly limited by its temporal extent. However, water isotope data from the past can be recovered in some areas from various sources, such as tree rings [29] and sediments [2]. By building the EVI-δ^18^O function and obtaining historical water isotope data, we could retrieve the paleo-EVI information. Since the time-series EVI can be an indicator for NPP [22], [30], we can then investigate the influences of paleoclimte on ecosystem productivity.

This study demonstrates that the EVI and water isotope data can compensate for each other and extend their applicability. The proposed approach can currently be applied to any terrestrial site with four precipitated δ^18^O data in a season. However, due to lacking of the isotopic data worldwide, the continental and global scale EVI-δ^18^O functions, such as incorporating latitudinal effects in different climate zones, cannot yet be achieved.. Therefore, our study highlights the importance of maintaining a comprehensive global isotope data network, such as improving the Global Network of Isotopes in Precipitation (GNIP), which facilitates constructing global water isoscapes at great detail.

## Supporting Information

Figure S1 **A local meteoric water line (b) (with corresponding residuals [a]) in the mountainous region of Taiwan.**
(TIF)

Figure S2 **Illustration of the integrated EVI.** The arrows point to the beginning and the end of a growing season depicted by the EVI time-series data. The shaded area is an example of the iEVI.
(TIF)

Figure S3 **Variablity of the EVI and δ^18^O.** The linear positive correlation between the variability of the EVI and the stability of δ^18^O. Only data from sites in natural forests were shown to demonstrate the relationship in nature habitats. However, the result is qualitatively the same if all sites were included.
(TIF)

Figure S4 **Summary of the prediction model construction process.** Yellow, blue and white boxes represent raw data needed for constructing the prediction model, procedures of building models, and predicted targets, respectively (i.e. Ingesting the iEVI of specific time and location in the model can predict δ^18^O and vice versa).
(TIF)

Figure S5 **A scatter plot of monthly predictions.** Monthly δ^18^O predictions vs. observations in regional scales.
(TIF)

Figure S6 **Predictabilities of the model across the elevation gradient.** Dots represent the mean predictabilities across an elevation gradient with standard errors (bars).
(TIF)

Table S1 **The integrated predictabilities (** ***r*** **±SEM [if shown]) under different initial conditions.**
(PDF**)**

Text S1 **Supplementary references.**
(PDF)
