# Supplementary figures and images for: Regional Scale High Resolution δ18O Prediction in Precipitation Using MODIS EVI
Source: PLoS One. 2012 Sep 19;7(9):e45496. doi: 10.1371/journal.pone.0045496 (PMC3446878; doi:10.1371/journal.pone.0045496)

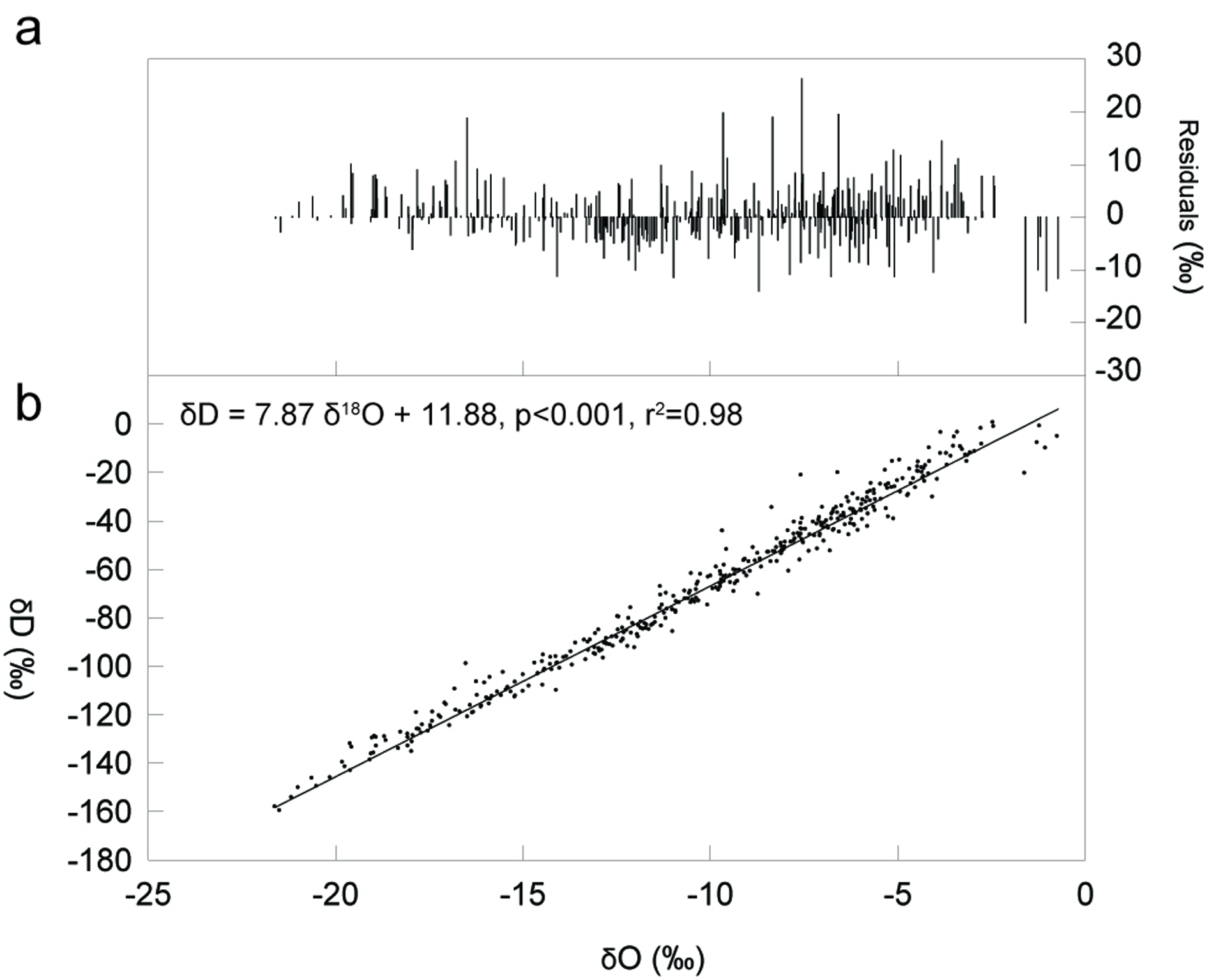

Supplement: Figure S1 — A local meteoric water line (b) (with corresponding residuals [a]) in the mountainous region of Taiwan. (TIF) [file pone.0045496.s001.tif]

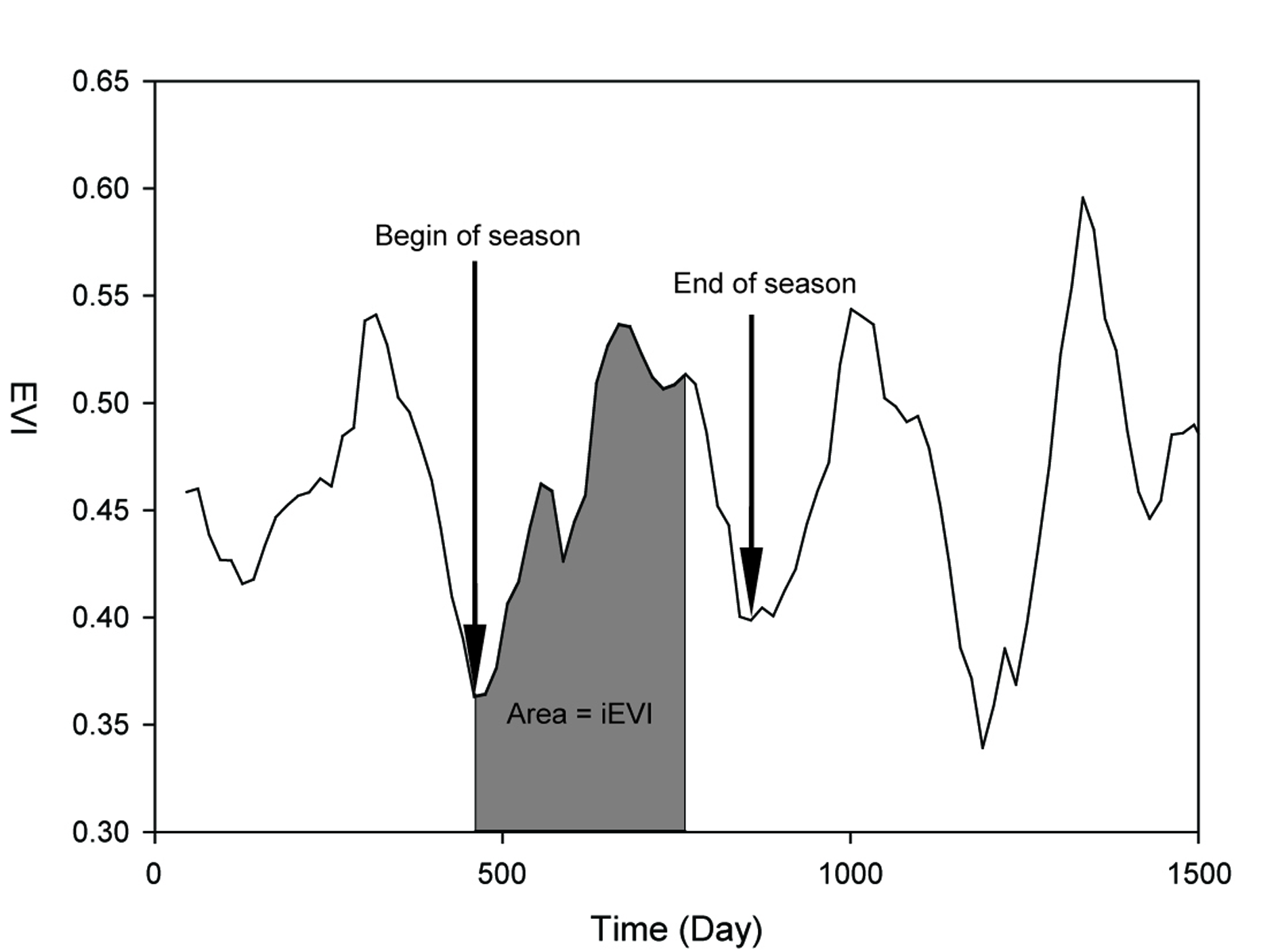

Supplement: Figure S2 — Illustration of the integrated EVI. The arrows point to the beginning and the end of a growing season depicted by the EVI time-series data. The shaded area is an example of the iEVI. (TIF) [file pone.0045496.s002.tif]

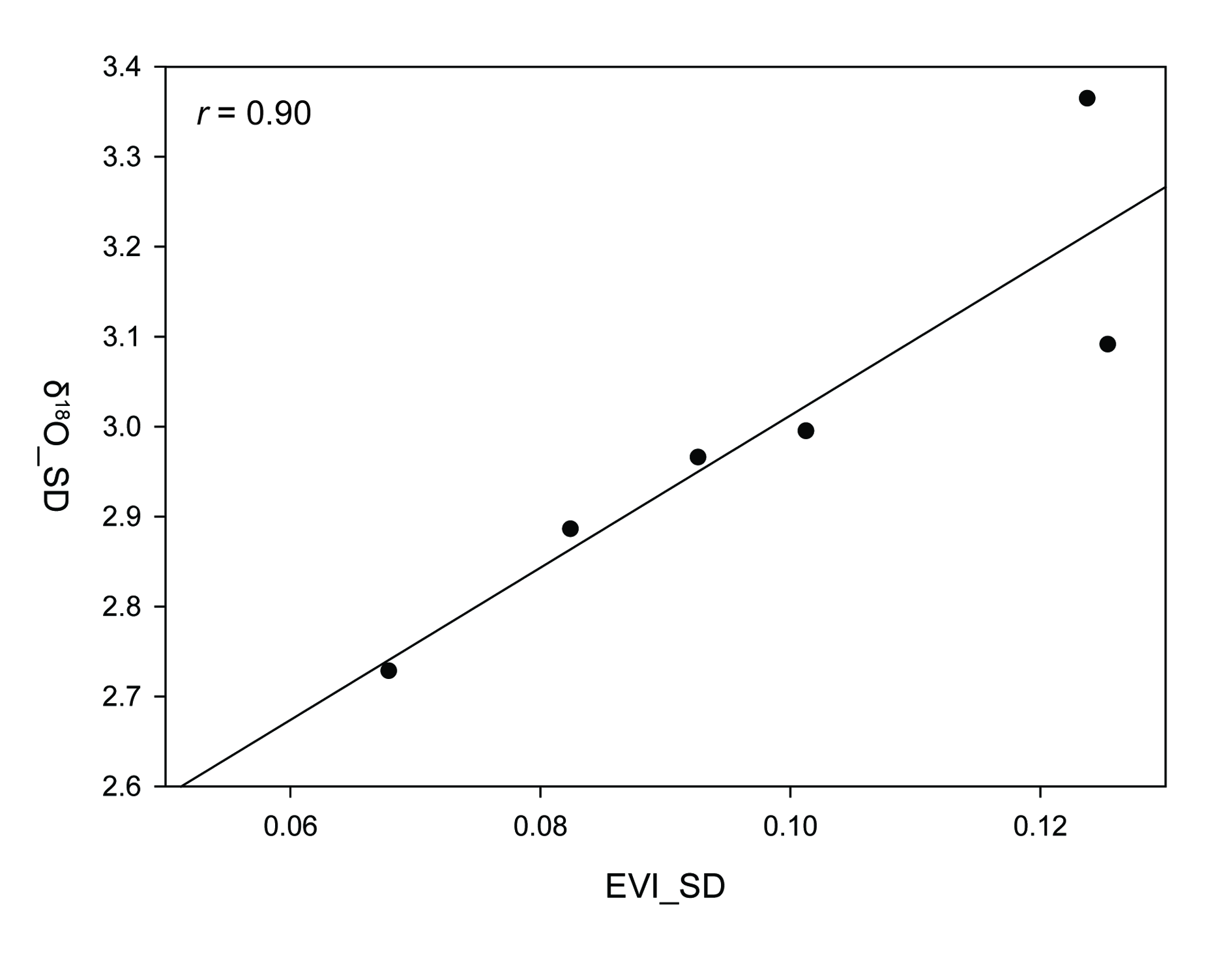

Supplement: Figure S3 — Variablity of the EVI and δ18O. The linear positive correlation between the variability of the EVI and the stability of δ18O. Only data from sites in natural forests were shown to demonstrate the relationship in nature habitats. However, the result is qualitatively the same if all sites were included. (TIF) [file pone.0045496.s003.tif]

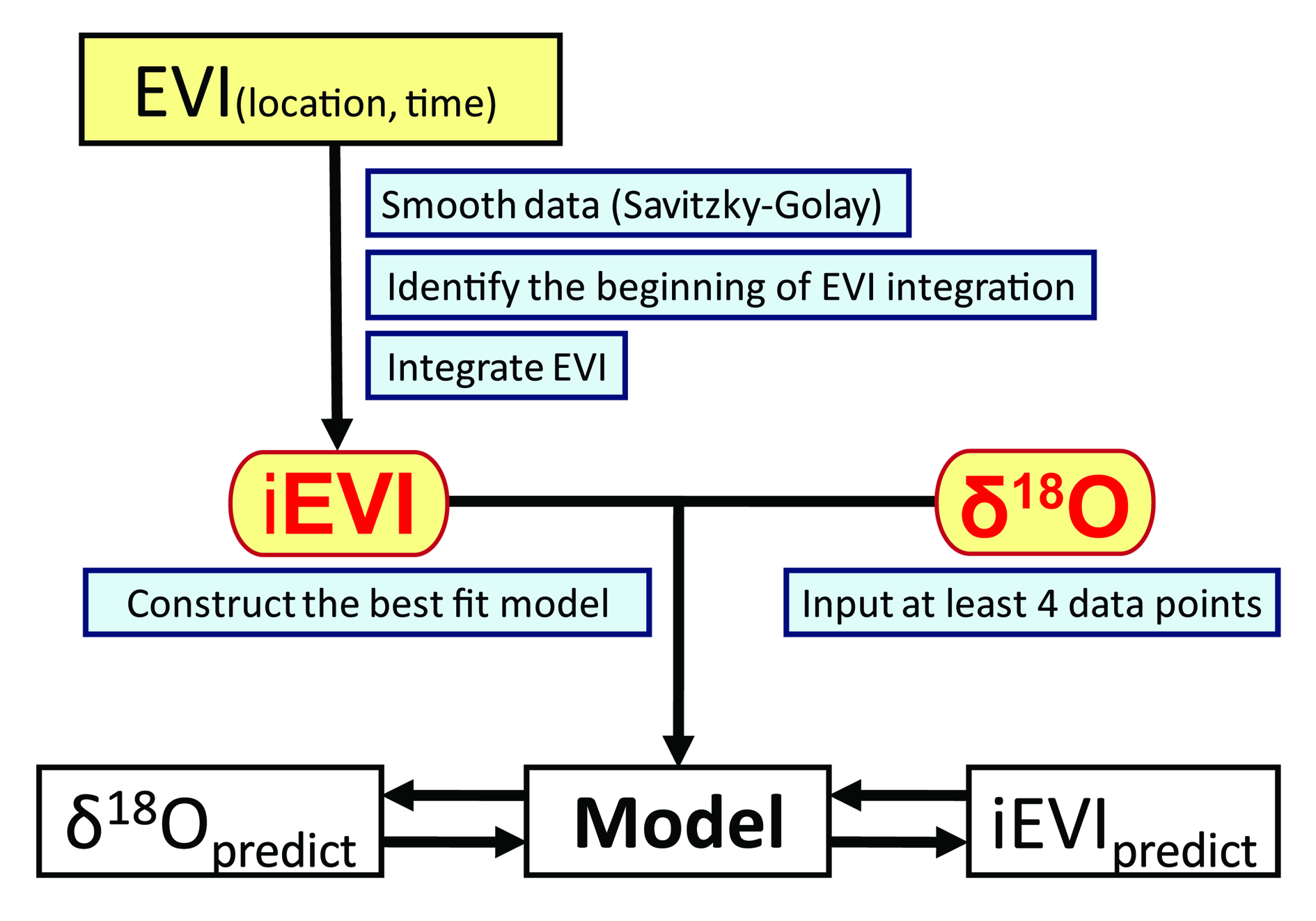

Supplement: Figure S4 — Summary of the prediction model construction process. Yellow, blue and white boxes represent raw data needed for constructing the prediction model, procedures of building models, and predicted targets, respectively (i.e. Ingesting the iEVI of specific time and location in the model can predict δ18O and vice versa). (TIF) [file pone.0045496.s004.tif]

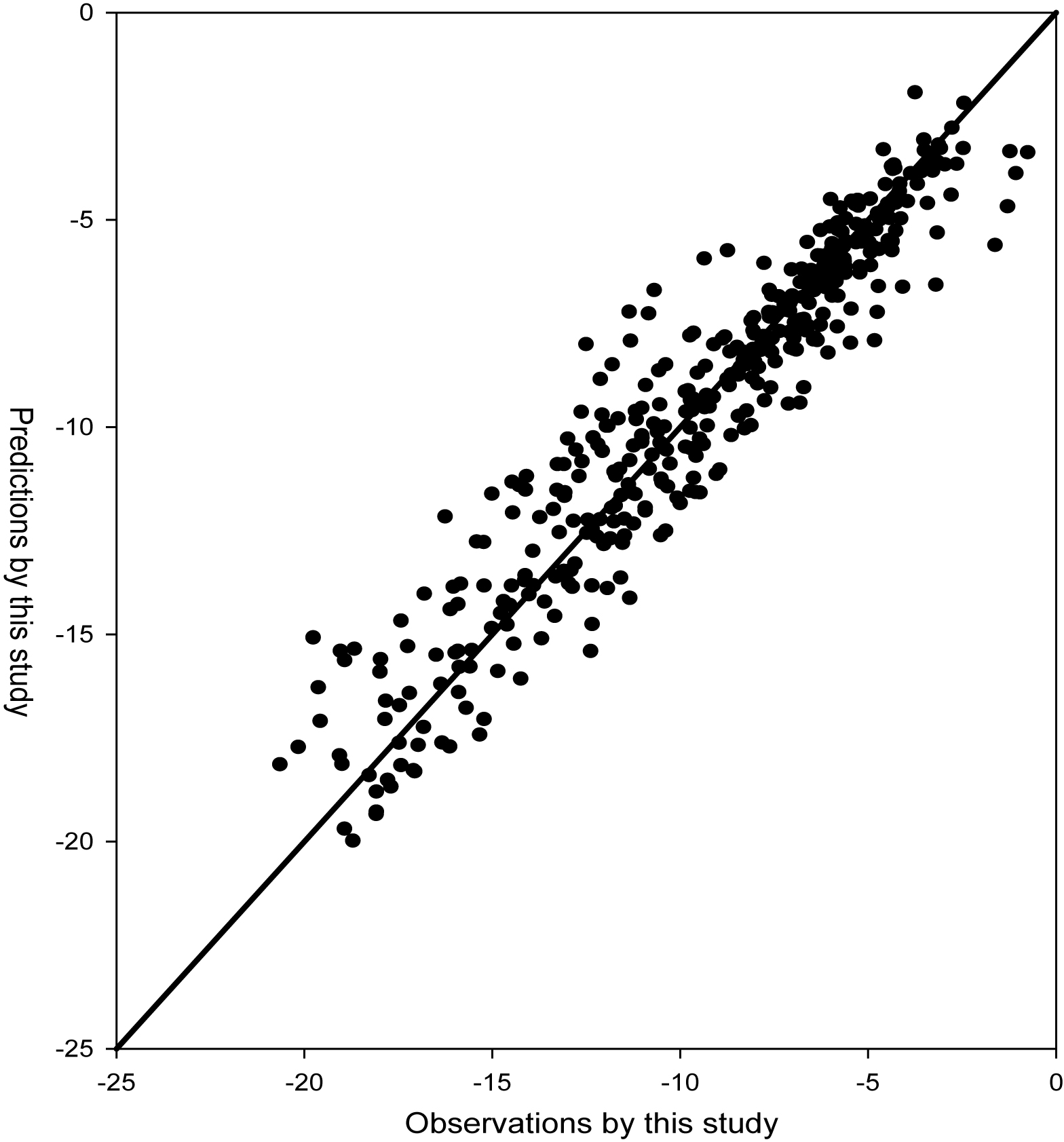

Supplement: Figure S5 — A scatter plot of monthly predictions. Monthly δ18O predictions vs. observations in regional scales. (TIF) [file pone.0045496.s005.tif]

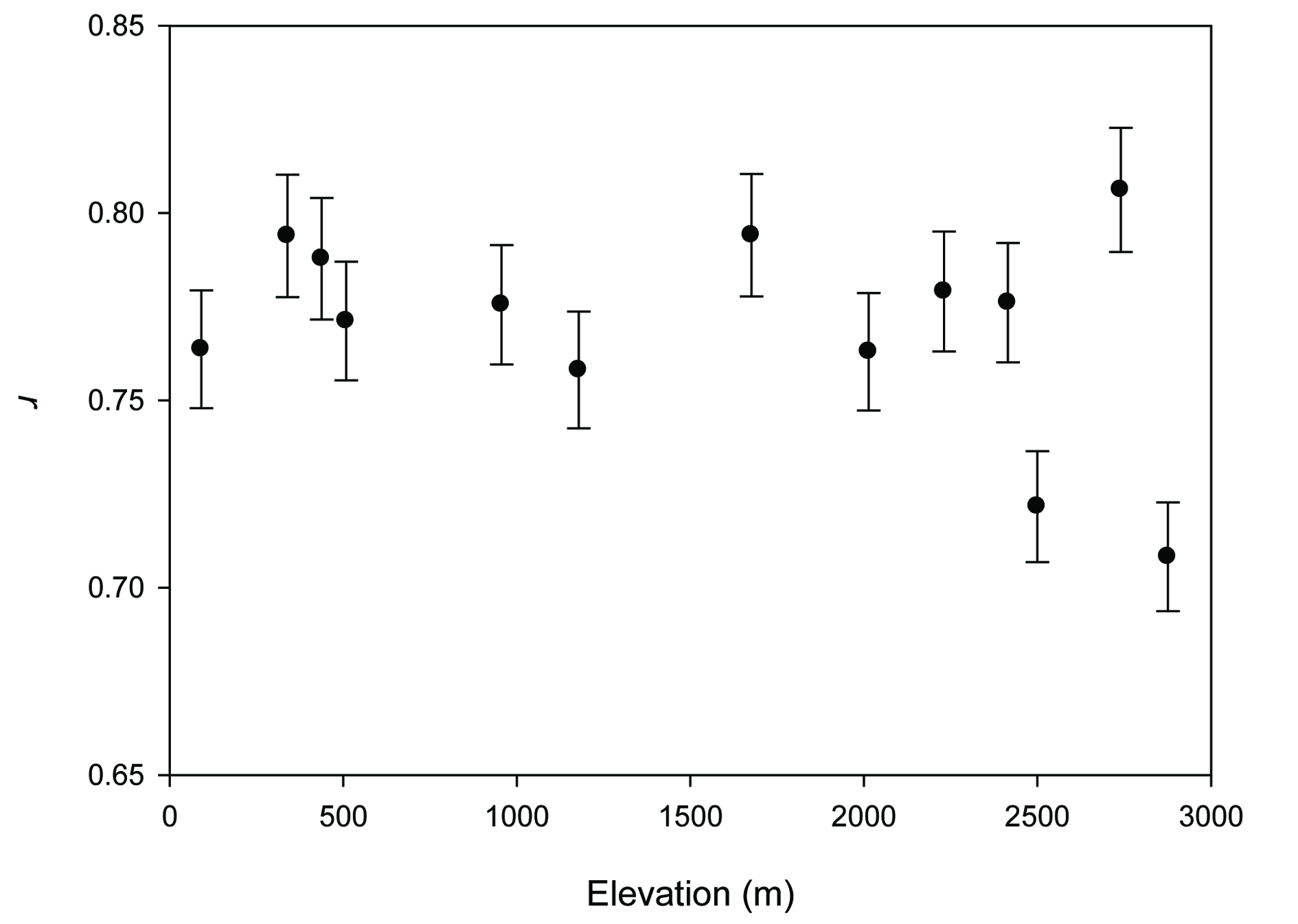

Supplement: Figure S6 — Predictabilities of the model across the elevation gradient. Dots represent the mean predictabilities across an elevation gradient with standard errors (bars). (TIF) [file pone.0045496.s006.tif]
