# Supplementary material for: Regional Scale High Resolution δ18O Prediction in Precipitation Using MODIS EVI
Source: PLoS One. 2012 Sep 19;7(9):e45496. doi: 10.1371/journal.pone.0045496 (PMC3446878; doi:10.1371/journal.pone.0045496)
Supplement: Table S1 — The integrated predictabilities ( r ±SEM [if shown]) under different initial conditions. (PDF) [file pone.0045496.s007.pdf]

Table S1 │The integrated predictabilities ($r \pm$ SEM [if shown]) under different initial conditions. The analysis is divided into three types: Type I: Using 4 paired data in 1 growing season to predict other data in other seasons; Type II: Using 4 paired data in 1 growing season to predict other data in the same growing seasons; Type III: Using 4 paired data in 1 growing season of 3 sites along elevation gradient to predict other data in other seasons at other altitudes in the same climate region.

Data source		Analysis					
		I		II		III	
		Annual	Monthly	Summer-Winter	Monthly	Annual	Monthly
Regional	13 sites	0.96	0.80±0.17	0.96	0.95±0.01	0.96	0.77±0.00
	Lishan		0.84				
	Piluchi		0.90				
