# Supplementary material for: Regional Scale High Resolution δ18O Prediction in Precipitation Using MODIS EVI
Source: PLoS One. 2012 Sep 19;7(9):e45496. doi: 10.1371/journal.pone.0045496 (PMC3446878; doi:10.1371/journal.pone.0045496)
Supplement: Text S1 — Supplementary references. (PDF) [file pone.0045496.s008.pdf]

Text S1

References

1. Gonfiantini R, Roche MA, Olivry JC, Fontes JC, & Zuppi GM (2001) The altitude effect on the isotopic composition of tropical rains. Chem. Geol. 181: 147-167.
2. Wang C-H & Peng T-R (2001) Hydrogen and oxygen isotopic compositions of Taipei precipitation: 1990 to 1998. West. Pac. Earth. Sci. 1: 429-442.
3. Bowen GJ & Wilkinson B (2002) Spatial distribution of delta O-18 in meteoric precipitation. Geology 30: 315-318.
4. Brown D (1994) Predicting vegetation types at treeline using topography and biophysical disturbance variables. J. Veg. Sci. 5: 641-656.
5. Holland PG & Steyn DG (1975) Vegetational Responses to Latitudinal Variations in Slope Angle and Aspect. J. Biogeogr. 2: 179-183.
6. Rech JA, Reeves RW, & Hendricks DM (2001) The influence of slope aspect on soil weathering processes in the Springerville volcanic field, Arizona. CATENA 43: 49-62.
7. Ricotta C & Avena GC (1997) The influence of meteorological conditions and topographic parameters on the beech forest microclimate of Simbruini Mountains, central Italy. Int. J. Remote Sens. 18: 505 - 516.
8. Bowen GJ, Wassenaar LI, & Hobson KA (2005) Global application of stable hydrogen and oxygen isotopes to wildlife forensics. Oecologia 143: 337-348.
9. Gat JR (1996) Oxygen and hydrogen isotopes in the hydrologic cycle. Annu. Rev. Earth. Planet. Sci. 24: 225-262.
10. Poage MA & Chamberlain CP (2001) Empirical relationships between elevation and the stable isotope composition of precipitation and surface waters: Considerations for studies of paleoelevation change. Amer. Jour. Sci. 301: 1-15.
11. Chen TC, Yen MC, Hsieh JC, & Arritt RW (1999) Diurnal and seasonal variations of the rainfall measured by the automatic rainfall and meteorological telemetry system in Taiwan. B Am Meteorol Soc 80: 2299-2312.
12. Dansgaard W (1964) Stable Isotopes in Precipitation. Tell 16: 436-468.
13. Bose JC (1920) Researches on growth of plants. Nature 105: 648-651.
14. Gallardo-Cruz J, Perez-Garcia EA, & Meave J (2009) beta-Diversity and vegetation structure as influenced by slope aspect and altitude in a seasonally dry tropical landscape. Landscape Ecol. 24: 473-482.
15. Myneni R, Keeling C, Tucker C, Asrar G, & Nemani R (1997) Increased plant growth in the northern high latitudes from 1981 to 1991. Nature 386:

698-702.
16. Van de Water P, Leavitt S, & Betancourt J (2002) Leaf d 13 C variability with elevation, slope aspect, and precipitation in the southwest United States. Oecologia 132: 332-343.
17. Whinam J, et al. (2003) A method for calculating environmental sensitivity to walker trampling in the Tasmanian Wilderness World Heritage Area. ELM: 151-165.
18. Huete AR, et al. (2006) Amazon rainforests green-up with sunlight in dry season. Geophys. Res. Lett. 33: L06405.
19. Silveira EMD, de Carvalho LMT, Acerbi-Junior FW, & de Mello JM (2008) The assessment of vegetation seasonal dynamics using multitemporal NDVI and EVI images derived from MODIS. Cerne 14: 177-184.
20. Zhang X, et al. (2003) Monitoring vegetation phenology using MODIS. Remote Sens. Environ. 84: 471-475.
21. Holdridge LR (1947) Determination of World Plant Formations from Simple Climatic Data. Science 105: 367-368.
22. Running S (1984) Microclimate control of forest productivity: analysis by computer simulation of annual photosynthesis/transpiration balance in different environments. Agr. Forest Meteoral. 32: 267-288.
23. Dongmann G (1974) Contribution of Land Photosynthesis to Stationary Enrichment of O-18 in Atmosphere. Radiat. Environ. Biophys. 11: 219-225.
24. Zundel G, Miekeley W, Grisi BM, & Forstel H (1978) (H2o)-O-18 Enrichment in Leaf Water of Tropic Trees - Comparison of Species from Tropical Rain-Forest and Semi-Arid Region in Brazil. Radiat. Environ. Biophys. 15: 203-212.
25. Allison G, Gat J, & Leaney F (1985) The relationship between deuterium and oxygen-18 delta values in leaf water. Chem. Geol. 58: 145-156.
